# NAD^+^ Synthetase is Required for Free-living and Symbiotic Nitrogen Fixation in the Actinobacterium *Frankia casuarinae*

**DOI:** 10.1264/jsme2.ME22093

**Published:** 2023-03-01

**Authors:** Ken-ichi Kucho, Koya Asukai, Thanh Van Nguyen

**Affiliations:** 1 Graduate School of Science and Engineering, Kagoshima University, 1–21–35 Korimoto, Kagoshima 890–0065, Japan

**Keywords:** *Casuarina*, forward genetics, NAD^+^ synthetase, nodule, suppressor mutant

## Abstract

*Frankia* spp. are multicellular actinobacteria that fix atmospheric dinitrogen (N_2_) not only in the free-living state, but also in root-nodule symbioses with more than 200 plant species, called actinorhizal plants. To identify novel *Frankia* genes involved in N_2_ fixation, we previously isolated mutants of *Frankia casuarinae* that cannot fix N_2_. One of these genes, mutant N3H4, did not induce nodulation when inoculated into the host plant *Casuarina glauca*. Cell lineages that regained the ability to fix N_2_ as free-living cells were isolated from the mutant cell population. These restored strains also regained the ability to stimulate nodulation. A comparative ana­lysis of the genomes of mutant N3H4 and restored strains revealed that the mutant carried a mutation (Thr584Ile) in the glutamine-dependent NAD^+^ synthetase gene (Francci3_3146), while restored strains carried an additional suppressor mutation (Asp478Asn) in the same gene. Under nitrogen-depleted conditions, the concentration of NAD(H) was markedly lower in the mutant strain than in the wild type, whereas it was higher in restored strains. These results indicate that glutamine-dependent NAD^+^ synthetase plays critical roles in both free-living and symbiotic N_2_ fixation in *Frankia*.

The genus *Frankia* is a multicellular member of the class *Actinobacteria*, order *Frankiales*, and family *Frankiaceae*. Most species in the genus *Frankia* fix atmospheric dinitrogen (N_2_), which is a unique property not found in any other genus of actinobacteria. N_2_ fixation occurs in spherical structures called vesicles, which are formed at the tips of hyphae. These vesicles are surrounded by several layers of hopanoid lipid envelopes that function as a barrier to oxygen (Berry *et al.*, 1993). Nitrogenase, an oxygen-labile enzyme, is exclusively produced in vesicles (Meesters, 1987).

*Frankia* establish root nodule symbioses with more than 200 plant species belonging to eight families, collectively known as actinorhizal plants (Benson and Dawson, 2007). Based on the phylogeny of their housekeeping genes, *Frankia* strains are classified into four lineages, each of which has a different host range (Pozzi *et al.*, 2018; Gtari *et al.*, 2019). Lineage 1 is divided into three subgroups (1a, 1b, and 1c). Lineages 1a and 1b infect plant species in Myricaceae and the genus *Alnus* of Betulaceae, while lineage 1c infects plant species in Casuarinaceae. Lineage 2 infects plant species in four families of the orders Rosales and Cucurbitales. Lineage 3 exhibits a broader host range, infecting plant species in several families of the orders Fagales and Rosales. Lineage 4 consists of atypical strains that cannot fix N_2_ or re-infect host plants.

Analyses of *Frankia* genomes identified various genes related to N_2_ fixation and symbiosis (Normand *et al.*, 2007; Tisa *et al.*, 2016). Genomes from lineages 1 to 3 contain 11 or 12 N_2_-fixation (*nif*) genes, including the structural genes encoding nitrogenase (*nifDKH*). All *Frankia* genomes contain genes that encode the biosynthetic pathway for hopanoid lipids. Genomes from lineage 2 *Frankia* contain homologues of the *nod* genes of rhizobia, which are involved in the synthesis of a symbiosis signaling molecule called Nod-factor (Persson *et al.*, 2015; Nguyen *et al.*, 2016, 2019).

Transcriptomic and proteomic approaches revealed numerous genes relevant to *Frankia* biology, such as nodulation (Alloisio *et al.*, 2010; Salgado *et al.*, 2018; Pujic *et al.*, 2022), sporulation (Béthencourt *et al.*, 2019), secondary metabolite biosynthesis (Udwary *et al.*, 2011), and responses to ammonium starvation (Alloisio *et al.*, 2007; Bickhart and Benson, 2011; Lurthy *et al.*, 2018), root exudates (Hammad *et al.*, 2001; Ktari *et al.*, 2017; Pujic *et al.*, 2019; Gueddou *et al.*, 2022), and salt stress (Oshone *et al.*, 2017). However, these studies lacked genetic evidence to demonstrate that these genes are truly involved in the relevant biological phenomena; *i.e.*, a mutation in a particular gene was not shown to disable the phenotype of interest.

To genetically identify novel *Frankia* genes involved in N_2_ fixation, we isolated mutants that were unable to fix N_2_ under free-living conditions (Kakoi *et al.*, 2014; Kucho *et al.*, 2017). Hyphae of *Frankia casuarinae* CcI3 were mutagenized with 1-methyl-3-nitro-1-nitrosoguanidine (NTG) or gamma rays, and approximately 50 mutants were isolated. These mutants did not exhibit detectable acetylene reduction activity (ARA) and did not grow under nitrogen-free (N–) conditions. They showed a wide variety of phenotypes related to vesicle development, including changes in their number, size, and envelope thickness (Kucho *et al.*, 2017; Asukai and Kucho, 2020). One of the mutants, strain N3H4, had apparently normal vesicle phenotypes with respect to number and size, but exhibited markedly reduced ARA (Kucho *et al.*, 2017). In the present study, we investigated the symbiotic phenotypes of mutant N3H4 and identified the genes responsible for aberrant phenotypes.

## Materials and Methods

### Bacterial strains and media

The wild-type (WT) strain CcI3 of *F. casuarinae* (Nouioui *et al.*, 2016), which is a symbiont of *Casuarina* and *Allocasuarina* plant species (Zhang *et al.*, 1984), and the N_2_-fixing mutant strain N3H4 (Kucho *et al.*, 2017) were used in the present study. The mutant strain N3H4 was isolated by mutagenesis with NTG.

BAP-T medium (Kucho *et al.*, 2009), which contains ammonium (5‍ ‍mM) as the main nitrogen source, was used to propagate *Frankia* cells under nitrogen-replete (N+) conditions. BAP-TN– medium, which is a derivative of BAP-T medium lacking ammonium, was used to induce N_2_-fixing ability under nitrogen-free (N–) conditions.

### Nodulation test

Seeds of *Casuarina glauca* were sterilized with 30% hydrogen peroxide for 15‍ ‍min, washed with sterilized distilled water (SDW), and germinated on 0.8% agar for one week. Plants were grown and nodulated in a hydroponic system as previously described (Alloisio *et al.*, 2010). Briefly, germinated seedlings were grown in vermiculite under fluorescent lighting with a 16-h light/8-h dark regime at 25°C for six weeks. They were transferred to Broughton and Dilworth’s medium (Broughton and Dilworth, 1971) in plastic pots and grown for four weeks with 5‍ ‍mM KNO_3_ and then for one week without KNO_3_. *Frankia* cells collected from 75‍ ‍mL of a late-exponential phase culture were inoculated into a pot. Four weeks after the inoculation, nodules were counted and ARA was measured.

### Isolation of strains with restored N_2_-fixing ability

Hyphae of mutant N3H4 were incubated in nitrogen-depleted CBminN– liquid medium (Kucho *et al.*, 2017) for 7 days to exhaust all intracellular nitrogen sources. Hyphae were collected from 15‍ ‍mL of the suspension by centrifugation (2,500×*g*, 20°C, 10‍ ‍min), washed twice with SDW, and resuspended in 2‍ ‍mL SDW. Hyphae were fragmented using the SoniMix ultrasonic homogenizer UX-050 (Mitsui Electric) with an output power setting of 50% for 10 s. The fragmented hyphal suspension was plated on solid CBminN– medium (100‍ ‍μL per dish) and incubated at 28°C for one month to obtain colonies. Single colonies were isolated, resuspended in SDW, streaked on solid CBminN– medium again, and incubated at 28°C for one month. This single colony isolation procedure was repeated three times. Cells were then propagated in BAP-T medium and their ability to fix N_2_ (as measured by the growth rate in N– liquid medium and ARA) was evaluated.

### Measurement of ARA

N_2_-fixing activity was evaluated as ARA. The nodulated root system of a *C. glauca* plant was detached and transferred to a test tube (2.4‍ ‍cm in diameter×12‍ ‍cm in height) and acetylene (5% [v/v]) was injected into the test tube. After a 2-h incubation at 25°C, 1‍ ‍mL of the gas phase was analyzed by gas chromatography (GC8-AIF; Shimadzu) to quantify the amount of ethylene generated.

The ARA of free-living *Frankia* cells was measured as previously described by Kucho *et al.* (2017). Briefly, *Frankia* hyphae precultured to the mid-exponential phase were collected by centrifugation (2,500×*g*, 20°C, 10‍ ‍min) and washed with BAP-TN– medium. Hyphae were inoculated into BAP-TN– medium (final OD_660_=0.02) and incubated at 28°C with stirring for 4 days. Five milliliters of the culture was transferred to a 7-mL vacutainer (BD Biosciences) and 5% (v/v) acetylene was injected. After a 4-h incubation at 28°C, 1‍ ‍mL of the gas phase was analyzed by gas chromatography (GC8-AIF, Shimadzu).

### Growth ana­lysis of liquid media

The growth of *Frankia* cells in N– liquid medium was analyzed as previously described (Kucho *et al.*, 2017). Briefly, *Frankia* hyphae precultured in BAP-T medium to the mid-exponential phase were collected by centrifugation and washed with BAP-TN– medium. Hyphae were homogenized by forced passage through a 21G needle and inoculated into BAP-TN– medium at an initial density of OD_660_=0.02. Cells were cultured with stirring at 28°C and cell density was continuously monitored using a self-made device constructed with the analog fiber sensor FX-11A (Panasonic Industrial Device) and the multichannel recorder MCR-4V (T and D).

### Genome ana­lysis

The genome sequencing and variant detection of restored strain r4 were performed at the Hubbard Center for Genome Studies (University of New Hampshire, Durham, NH, USA) using the Illumina HiSeq2500 platform according to the procedure described by Kucho *et al.* (2017). The genome sequencing and variant detection of restored strains r8, r9, and r10 were performed by BGI genomics using the Illumina HiSeq4000 platform.

### Prediction of the protein tertiary structure

The predicted tertiary structure of the Francci3_3146 protein was obtained from the Uniprot database (https://www.uniprot.org/) with the accession number Q2J889.

### Alignment ana­lysis

An alignment ana­lysis was performed using the Clustal Omega program at the Uniprot Align site (https://www.uniprot.org/align). The microbial sources of the deduced amino acid sequences of NAD^+^ synthetases in the alignment are shown in Table S1.

### Measurement of the intracellular concentration of NAD(H)

*Frankia* cells were propagated in 150‍ ‍mL of BAP-T medium to the mid-exponential phase. One hundred milliliters of culture was collected as the N+ cell sample. The remaining cells were acclimated to N– conditions according to the procedure for measuring ARA (see above) in 150‍ ‍mL BAP-TN– medium. Cells were collected by centrifugation (2,500×*g*, 20°C, 10‍ ‍min), resuspended in PBS buffer (8.1‍ ‍mM Na_2_HPO_4_, 1.47‍ ‍mM KH_2_PO_4_, 137‍ ‍mM NaCl, and 2.7‍ ‍mM KCl, pH 7.4), and disrupted using the SoniMix ultrasonic homogenizer UX-050 (Mitsui Electric). Cell debris was removed by centrifugation (22,000×*g*, 4°C, 10‍ ‍min) and the supernatant was used to quantify NAD(H). The amount of NAD(H) was measured using the Amplite colorimetric total NAD and NADH assay kit (AAT Bioquest) by monitoring absorbance at 575‍ ‍nm. The protein concentrations of lysates were assessed using a protein assay dye reagent (Bio-Rad). The concentration of NAD(H) in the lysates was represented as the amount of NAD(H) (pmol) µg^–1^ protein.

## Results

### Symbiotic phenotypes of mutant N3H4

When WT *F. casuarinae* was used to inoculate the host plant *C. glauca*, it induced nodulation in all of the individual plants tested and the average number of nodules was 55 plant^–1^ (Table 1). Nodulated plants exhibited high ARA. In contrast, plants inoculated with mutant N3H4 did not form any visible nodules and exhibited markedly reduced ARA (Table 1). These results indicate that mutant N3H4, which is defective under free-living N_2_ fixation (Kucho *et al.*, 2017), did not form a symbiotic association with the host plant.

### Isolation of strains with restored N_2_-fixing ability

**Fig. 1.**
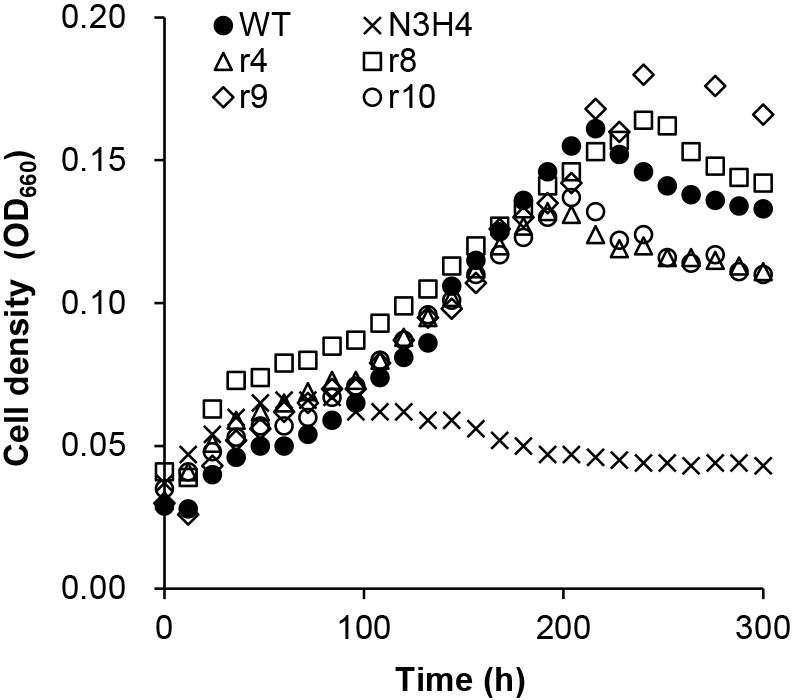
Growth of *Frankia* strains in N– liquid medium. WT, mutant N3H4, and restored strains (r4, r8, r9, and r10) were inoculated into N– liquid medium and the OD_660_ of cultures was measured every 12 h.

**Fig. 2.**
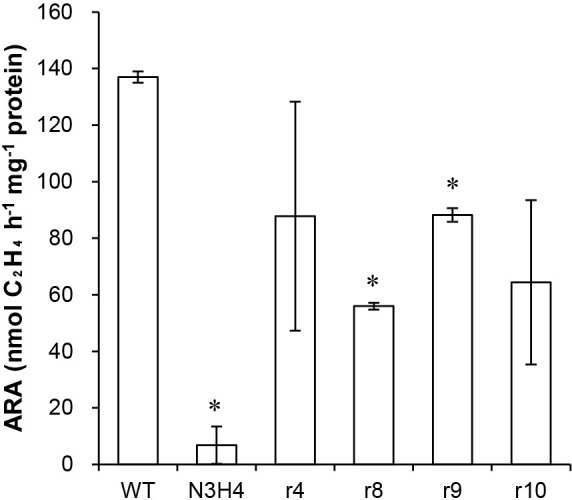
Acetylene reduction activity of *Frankia* strains under free-living conditions. Averages (open boxes) and SD (bars) from two to four biological replicates are shown. Asterisks indicate a significant difference from WT (the *t*-test,* P*<0.05).

The cells of mutant N3H4 had been subcultured in liquid medium for a few years. Therefore, spontaneous second mutations may have occurred in the chromosomes of some cells due to erroneous DNA replication. If a second mutation occurs at the mutated locus in the gene required for N_2_ fixation and regenerates the original wild-type sequence, the cell regains N_2_-fixing ability (reversion). Alternatively, a second mutation may occur elsewhere in the gene that compensates for the deleterious effects of the first mutation (suppressor mutation). To isolate revertants and suppressor mutants (hereafter referred to as restored strains), mutant N3H4 cells were cultivated on N– solid medium. When 3×10^7^ colony-forming units (CFU) were inoculated, seven colonies appeared. Four of these colonies (strains r4, r8, r9, and r10) were characterized in detail. In N– liquid medium, the four restored strains grew at a similar rate to the WT, while mutant N3H4 showed markedly reduced growth (Fig. 1). As expected, the restored strains exhibited significantly higher ARA than mutant N3H4 (Fig. 2). These results indicate that strains r4, r8, r9, and r10 regained free-living N_2_-fixing ability.

When the four restored strains were used to inoculate *C. glauca* plants, they induced nodulation on all of the individual plants tested, and the average number of nodules was similar to that in the plants inoculated with WT *F. casuarinae* (Table 1). In addition, plants infected with the restored strains exhibited similar ARA to those infected with WT (Table 1). Therefore, the restored strains simultaneously regained free-living and symbiotic N_2_-fixing abilities. This result suggests that the gene impaired in mutant N3H4 was critical for both types of N_2_ fixation.

### Genome ana­lysis

A total of 110 mutations were previously detected in the mutant N3H4 genome (Kucho *et al.*, 2017). Among them, 54 mutations altered amino acid sequences (Table S2); therefore, any of these mutations may be responsible for the mutant phenotypes of strain N3H4. To identify the mutation responsible, we sequenced the genomes of the four restored strains. Forty-four out of the 54 mutations were detected in the genomes of all four restored strains (Table S2). The remaining 10 mutations were also detected in strain r4, indicating that reversion had not occurred. However, several of the 10 mutations were not detected in strain r8, r9, or r10 (Table S2, yellow highlight). There were 9 undetected mutations in strain r8, 7 in strain r9, and 7 in strain r10. This result suggests that these mutation sites reverted to the wild-type sequence. However, since reversion is a very rare event, it is unlikely that so many (between 7 and 9) reversions occurred in a single strain. Therefore, these strains may actually have carried these mutations; however, our next-generation sequencing ana­lysis did not detect them. The read depth of sequence data was significantly lower in strains r8, r9, and r10 (approximately 200×) than in strain r4 (1,700×), which may be the reason for the larger number of undetected mutations in the genomes of strains r8, r9, and r10.

**Fig. 3.**
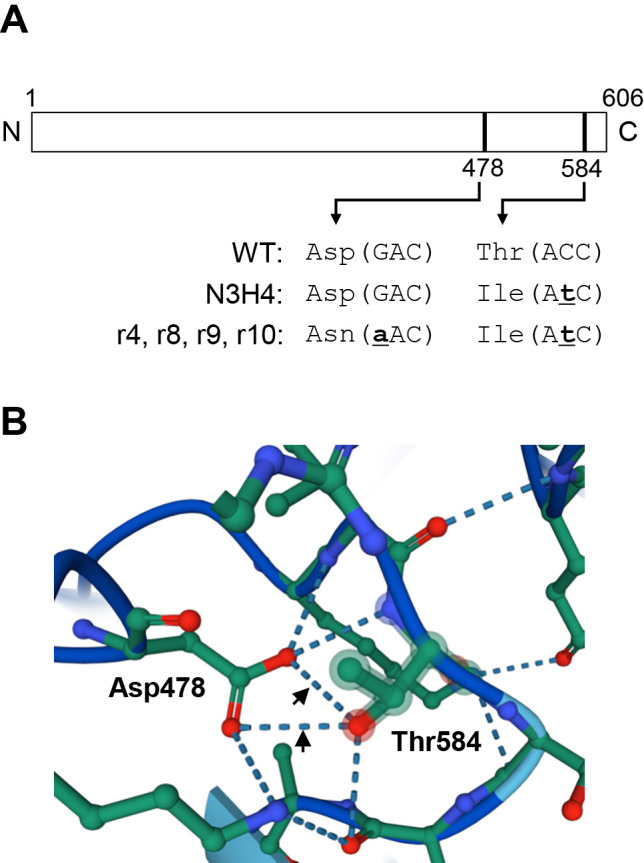
Structure of the Francci3_3146 protein. (A) Schematic diagram of the primary structure of the protein. Numbers represent the positions of amino acids. (B) Predicted tertiary structure. Arrowheads indicate possible hydrogen bonding. Red, blue, and green balls represent atoms of oxygen, nitrogen, and carbon, respectively. Hydrogen atoms are not shown.

Furthermore, we found a putative intragenic suppressor mutation in the Francci3_3146 gene that coded for NAD^+^ synthetase (Table S2, blue highlight). In mutant N3H4, the 584th amino acid residue (Thr; ACC) in the Francci3_3146 gene was mutated to Ile (AtC) (Fig. 3A and Table S2, orange highlight). All four restored strains carried this mutation. Additionally, they all carried a second mutation in the same gene; the 478th residue (Asp; GAC) was mutated to Asn (aAC) (Fig. 3A and Table S2, blue highlight). These mutations were confirmed by the Sanger method (data not shown). Although the two amino acids (Asp478 and Thr584) were separated by 106 amino acid residues in the primary structure, they were located in close proximity in the predicted tertiary structure and formed hydrogen bonds (Fig. 3B, arrowheads). These results suggest that the substitution at position 584 (Thr584Ile) was the mutation that inactivated this enzyme, while the substitution at position 478 (Asp478Asn) was a suppressor mutation that occurred in the restored strains.

### Francci3_3146 encodes a glutamine-dependent NAD^+^ synthetase

NAD^+^ synthetases catalyze the following reaction: nicotinic acid adenine dinucleotide (NaAD)+amino group donor+ATP→NAD^+^+AMP+PP_i_ (Zalkin, 1985). NAD^+^ synthetases are classified into two types based on amino group donors and protein structures. Ammonia-dependent NAD^+^ synthetases require ammonia as the sole amino group donor and are found in many Bacteria and Archaea (de Ingeniis *et al.*, 2012). Glutamine-dependent NAD^+^ synthetases utilize glutamine and ammonia as amino group donors and are found in all Eukaryotes, many Bacteria, and a few Archaea (de Ingeniis *et al.*, 2012). Glutamine-dependent enzymes have a glutaminase domain, which generates ammonia by the hydrolysis of glutamine; therefore, the polypeptide (*ca.* 600 amino acids) is longer than that of ammonia-dependent enzymes (*ca.* 300 amino acids).

An alignment of the deduced amino acid sequence of Francci3_3146 with those of known NAD^+^ synthetases is shown in Fig. S1. Francci3_3146 showed high amino acid sequence identities (30–60%) with the glutamine-dependent NAD^+^ synthetases from *Streptomyces avermitilis* (Sav), *Rhodobacter capsulatus* (Rca), *Thermotoga maritima* (Tma), and *Mycobacterium tuberculosis* (Mtu), and had a‍ ‍glutaminase domain (Fig. S1, orange line) that was absent‍ ‍in the ammonia-dependent enzyme from *Salmonella typhimurium* (Sty). Most of the important amino acid residues, such as the binding sites for glutamine, NaAD, and‍ ‍ATP, were conserved in the Francci3_3146 protein. In‍ ‍addition, Asp at position 478 in Francci3_3146 (Fig. S1, red box) was conserved in the other bacterial enzymes, suggesting an important function. These results indicate that Francci3_3146 encodes a glutamine-dependent NAD^+^ synthetase. Homology searches suggested that Francci3_3164 was the only NAD^+^ synthetase gene present in the genome of *F. casuarinae* CcI3 (data not shown).

### Intracellular concentration of NAD(H)

**Fig. 4.**
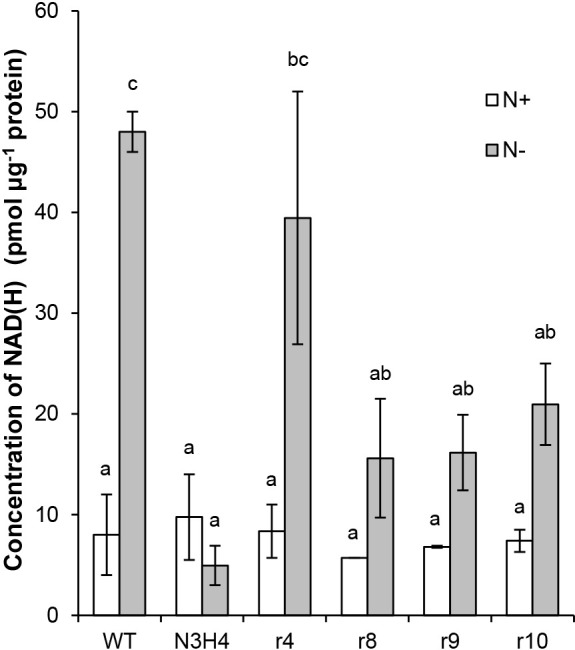
Intracellular concentrations of NAD(H) in *Frankia* strains. Averages (open boxes) and SD (bars) from two biological replicates are shown. Open and gray boxes represent N+ and N– conditions, respectively. Different letters indicate significant differences between conditions (the Tukey-Kramer test, *P*<0.05).

NAD^+^ is reduced to NADH by various enzymatic pathways, including the tricarboxylic acid (TCA) cycle. To investigate whether the mutation (Thr584Ile) in the Francci3_3164 gene inactivated the enzymatic activity of the protein, we measured the total intracellular concentration of NAD^+^ and NADH (NAD(H)). In the WT strain, the concentration of NAD(H) was markedly higher under N– conditions than under N+ conditions (Fig. 4), suggesting the promotion of NAD(H) synthesis under N_2_-fixing conditions. In mutant N3H4, the concentration of NAD(H) was similar to that in the WT under N+ conditions, but was markedly lower under N– conditions (Fig. 4), indicating a defect in the ability to synthesize NAD^+^. As expected, under N– conditions, the concentrations of NAD(H) were higher in all restored strains than in mutant N3H4, indicating that the ability to synthesize NAD^+^ was restored. Under N+ conditions, these restored strains showed similar NAD(H) concentrations to those in the WT and mutant N3H4. These results indicate that the decrease in the ability to synthesize NAD^+^ under N– conditions caused the N_2_-fixing defect in the N3H4 mutant.

## Discussion

In the present study, we reported that mutant N3H4 was defective not only for free-living N_2_ fixation (Kucho *et al.*, 2017), but also for stimulating nodulation in the host plant *Casuarina* (Table 1). Since the nodulation test was conducted in nitrogen-free medium using a hydroponic cultivation system (see Materials and Methods), the inability to carry out free-living N_2_ fixation may have affected the efficiency of nodulation. It takes at least two to three weeks from the inoculation of *Frankia* to the formation of nodules. The mutant may not have been able to survive under nitrogen-free conditions for such a long time. A growth ana­lysis in N– medium showed that mutant N3H4 shifted to the death phase approximately 150‍ ‍h (6 to 7 days) after the start of the cultivation (Fig. 1).

In the present study, we showed that the glutamine-dependent NAD^+^ synthase gene (Francci3_3146) played a critical role in N_2_ fixation in *F. casuarinae* based on three lines of evidence, the first of which is genetic evidence. In the WT Francci3_3146 protein, the hydroxyl group of Thr584 was predicted to form hydrogen bonds with the carboxyl group of Asp478 (Fig. 3B). In the mutant protein, Thr584 was substituted by Ile584 (Fig. 3A), which has a hydrophobic side chain without a hydroxyl group. Therefore, Ile did not form a hydrogen bond with Asp478 and may also repel its carboxyl group, which may destabilize the folding of the polypeptide and reduce its enzymatic activity. In the restored strains, Asp478 was substituted by Asn478 (Fig. 3A). Since Asn is less polar than Asp, repulsion with hydrophobic Ile584 may be alleviated; therefore, the stability of the folded polypeptide may be increased and enzymatic activity may be restored. The detailed X-ray crystal structure of a glutamine-dependent NAD^+^ synthetase was‍ ‍reported for the actinobacterium *M. tuberculosis* (Chuenchor *et al.*, 2012). In its structure, Gly507, which corresponds to Asp478 of the *F. casuarinae* enzyme (Fig. S1), and Ser646 (corresponding to Thr584 of the *F. casuarinae* enzyme) were located in close proximity to the ATP- and NaAD-binding sites (Fig. S2). Therefore, the loss of the interaction between the two amino acids may negatively affect substrate binding and reduce enzymatic activity.

The second piece of evidence is based on biochemical data. As expected from the genetic data, mutant N3H4 showed a significantly lower concentration of NAD(H) under N– conditions (Fig. 4). Moreover, in all of the restored strains, intracellular NAD(H) was restored to higher concentrations than in the mutant, although not as high as in the WT (Fig. 4). N_2_ fixation is a reaction that consumes large amounts of energy, requiring as many as 16‍ ‍molecules of ATP to fix one N_2_ molecule (Dixon and Kahn, 2004). *Frankia*, an aerobic bacterium, synthesizes the ATP required for N_2_ fixation through respiration. Respiration utilizes NADH as an electron donor, which is generated by the reduction of NAD^+^ in the TCA cycle. Consistent with this scenario, a significantly larger amount of NAD(H) was synthesized under N– conditions than under N+ conditions in the WT (Fig. 4). In contrast to WT cells, mutant N3H4 cells contained a markedly reduced concentration of NAD(H), which may be attributed to a mutation (Thr584Ile) in the NAD^+^ synthetase gene Francci3_3146 (Fig. 3A). The reduced concentration of NAD(H) may decrease ATP synthesis during respiration, which may in turn inhibit N_2_ fixation.

The concentration of NAD(H) in WT was significantly lower under N+ conditions than under N– conditions (Fig. 4), which may reflect the lower demand for the compounds. In all strains, including mutant N3H4, the concentrations of NAD(H) were similar to that in the WT (Fig. 4, open box). Under N+ conditions, the N3H4 mutant grew at a similar rate as the WT (Kucho *et al.*, 2017), which appeared to be due to mutant NAD^+^ synthase synthesizing the low amount of NAD^+^ required under these conditions.

The third line of evidence is based on previous findings. In other bacteria, mutations in NAD^+^ synthetase genes also resulted in similar phenotypes to those observed in *F. casuarinae*. A *S. typhimurium* mutant with a mutation in the NAD^+^ synthetase gene poorly grew at low concentrations (<1‍ ‍mM) of ammonium (Broach *et al.*, 1976; Schneider and Reitzer, 1998). Another study isolated several N_2_-fixing mutants of *R. capsulatus*, which is a free-living diazotrophic bacterium (Willison *et al.*, 1985). One of the mutants (strain RC34) did not grow under nitrogen-free conditions (Zinchenko *et al.*, 1990) and did not exhibit nitrogenase activity (Willison *et al.*, 1985). A genetic complementation test demonstrated that the gene responsible for these aberrant phenotypes encoded NAD^+^ synthetase (Willison and Tissot, 1994).

In conclusion, by making use of mutant N3H4 and its derivative suppressor strains, we demonstrated that a glutamine-dependent NAD^+^ synthetase gene (Francci3_3146) was required for free-living and symbiotic N_2_ fixation by *F. casuarinae*. The failure of a plant to form nodules may be a secondary effect of a defect in free-living N_2_ fixation. To the best of our knowledge, this is the first study to identify the function of a *Frankia* gene using a forward genetic approach. By applying this strategy to other mutants, we will be able to identify novel genes involved in *Frankia*-specific properties, such as vesicle differentiation.

## Citation

Kucho, K., Asukai, K., and Nguyen, T. V. (2023) NAD^+^ Synthetase is Required for Free-living and Symbiotic Nitrogen Fixation in the Actinobacterium *Frankia casuarinae*. *Microbes Environ ***38**: ME22093.

https://doi.org/10.1264/jsme2.ME22093

## Supplementary Material

Supplementary Material

## Figures and Tables

**Table 1.** Symbiotic phenotypes of WT and mutant *Frankia* strains

Inoculant strain	Nodulation				N_2_ fixation	
	Number of plants		Nodules plant^–1a^		ARA^a,b^	Number of plants
	Tested	Nodulated				
Wild type	13	13	55±12		212±98	8
N3H4	23	0	0*		1.2±0.9*	19
r4	6	6	41±11		92±47	6
r8	5	5	85±24		238±85	5
r9	6	6	80±18		237±80	6
r10	5	5	70±12		404±112	5
None	7	0	0*		0.1±0.1	6

^a^ Average±SE, ^b^ nmol ethylene plant^–1^ hr^–1^. Asterisks indicate a significant difference from the wild type (the *t*-test,* P*<0.05).
