# Supplementary material for: NAD+ Synthetase is Required for Free-living and Symbiotic Nitrogen Fixation in the Actinobacterium Frankia casuarinae
Source: Microbes Environ. 2023 Mar 1;38(1):ME22093. doi: 10.1264/jsme2.ME22093 (PMC10037102; doi:10.1264/jsme2.ME22093)
Supplement: Supplementary file 1 — Supplementary Material [file 38_22093_s1.pdf]

Table S1. Source of amino acid sequences of NAD^+ synthetase used for the alignment analysis.

Bacterial species	Type	Length (a.a.)	Uniprot accession No.	Reference
Frankia causarinae	Glutamine-dependent	606	Q2J889	Normand *et al.*, 2007
Streptomyces avermitilis	Glutamine-dependent	584	Q82AV7	Ikeda *et al.*, 2003
Rhodobacter capsulatus	Glutamine-dependent	552	Q03638	Willison and Tissot, 1994
Thermotoga maritima	Glutamine-dependent	576	Q9X0Y0	Resto *et al.*, 2009
Mycobacterium tuberculosis	Glutamine-dependent	679	P9WJJ3	Rizzi *et al.*, 1998
Salmonella typhimurium	Ammonia-dependent	275	Q8ZPU5	McClelland *et al.*, 2001

Table S2. Mutations in the mutant N3H4 genome that changed the amino acid sequence. A suppressor mutation found in the restored strains is also shown (blue highlight).

Position (nt)	WT	N3H4	r4	r8	r9	r10	Gene ID	Annotation	Amino acid change
3,774	C	T	m [a]	m	m	m	Francci3_0004	Recombination protein F	Ala26Val
29,901	C	T	m	ND [b]	m	m	Francci3_0021	CRISPR-associated Cas5e family protein	Pro232Ser
30,413	C	T	m	m	ND	m	Francci3_0022	Hypothetical protein	Thr123Ile
90,047	C	T	m	m	m	m	Francci3_0071	Hypothetical protein	Pro223Ser
344,203	G	A	m	ND	ND	ND	Francci3_0296	Insertion element hypothetical protein	Pro198Leu
374,084	C	T	m	m	m	m	Francci3_0320	Periplasmic sensor signal transduction histidine kinase	Glu407Lys
491,451	C	T	m	m	m	m	Francci3_0412	Cell division FtsK/SpoIIIE	Arg143Gln
936,706	C	T	m	m	m	m	Francci3_0804	Hypothetical protein	Arg2Gln
1,022,313	G	A	m	m	m	m	Francci3_0877	DNA integration/recombination/inversion protein	Ala115Thr
1,155,430	C	T	m	ND	m	ND	Francci3_0976	NAD-dependent epimerase/dehydratase	Val70Ile
1,168,624	C	T	m	m	m	m	Francci3_0988	Carboxyl transferase	Glu596Lys
1,278,183	CGCCCGGTC	-	m	m	m	m	Francci3_1072	(NiFe) hydrogenase maturation protein HypF	Deletion
1,411,329	C	T	m	m	m	m	Francci3_1180	Exodeoxyribonuclease III	Ala286Thr
1,574,610	C	T	m	m	m	m	Francci3_1312	Aconitase	Ala809Val
1,585,964	G	A	m	ND	ND	m	Francci3_1323	TrkA-like	Thr85Ile
1,769,207	C	T	m	m	m	m	Francci3_1475	Cell division FtsK/SpoIIIE	Arg982Trp
1,841,850	CGCTGCCGCCGTT	-	m	m	m	m	Francci3_1535	ATPase	Frameshift
1,971,434	C	T	m	m	m	m	Francci3_1638	Phosphoglycerate kinase	Thr285Ile
1,991,103	T	C	m	m	m	m	Francci3_1657	Glycerol kinase	Ser414Gly

Position (nt)	WT	N3H4	r4	r8	r9	r10	Gene ID	Annotation	Amino acid change
2,140,051	C	T	m	m	m	m	Francci3_1805	Hypothetical protein	Val340Ile
2,140,329	C	T	m	m	m	m	Francci3_1805	Hypothetical protein	Gly247Asp
2,141,118	C	T	m	m	m	m	Francci3_1806	AMP-dependent synthetase and ligase	Val510Ile
2,200,738	G	A	m	m	m	m	Francci3_1865	Hypothetical protein	Trp10Stop
2,337,453	C	T	m	m	m	m	Francci3_1989	Putative PAS/PAC sensor protein	Gly252Asp
2,366,992	GCGACCGC CCGGGAC	-	m	ND	ND	ND	Francci3_2015	MarR family transcriptional regulator	Deletion
2,456,458	C	T	m	ND	ND	ND	Francci3_2088	Recombinase	Ala463Val
2,484,710	C	T	m	m	m	m	Francci3_2118	Oligopeptide/dipeptide ABC transporter, ATP-binding protein-like	Ala131Val
2,485,624	C	T	m	m	m	m	Francci3_2118	Oligopeptide/dipeptide ABC transporter, ATP-binding protein-like	Pro436Ser
2,649,580	G	A	m	m	m	m	Francci3_2272	Apolipoprotein N-acyltransferase	Ala381Val
2,650,276	G	A	m	m	m	m	Francci3_2272	Apolipoprotein N-acyltransferase	Pro149Leu
2,892,325	C	T	m	m	m	m	Francci3_2461	Amino acid adenylation	Trp3215Stop
2,898,345	C	T	m	ND	ND	ND	Francci3_2461	Amino acid adenylation	Ala1209Thr
2,914,160	G	A	m	m	m	m	Francci3_2468	Carbon starvation protein CstA	Ala201Val
3,434,839	G	A	m	m	m	m	Francci3_2915	Hypothetical protein	Ala370Val
3,589,696	C	T	m	m	m	m	Francci3_3023	Phosphoribosyl isomerase A	Ala37Thr
3,724,379	G	G	A	A	A	A	Francci3_3146	NAD^+ synthetase	Asp478Asn
3,724,698	C	T	m	m	m	m	Francci3_3146	NAD^+ synthetase	Thr584Ile
3,855,625	C	T	m	m	m	m	Francci3_3256	WD-40 repeat-containing serine/threonine protein kinase	Gly758Asp

Position (nt)	WT	N3H4	r4	r8	r9	r10	Gene ID	Annotation	Amino acid change
3,863,591	G	A	m	m	m	m	Francci3_3259	Glycoside hydrolase family protein	Glu313Lys
3,915,655	C	T	m	m	m	m	Francci3_3305	Type IV secretory pathway VirD4 components-like	Ala238Thr
3,916,878	C	T	m	m	m	m	Francci3_3306	Hypothetical protein	Ala126Thr
3,917,772	C	T	m	m	m	m	Francci3_3307	Zinc finger, CHC2-type	Ala33Val
3,996,291	G	A	m	m	m	m	Francci3_3367	Hypothetical protein	Pro154Leu
4,163,452	G	A	m	m	m	m	Francci3_3492	Polyphosphate glucokinase	Asp22Asn
4,324,078	G	A	m	m	m	m	Francci3_3621	Isopropyl malate isomerase large subunit	Pro299Ser
4,398,993	G	A	m	m	m	m	Francci3_3671	Hypothetical protein	Pro272Leu
4,453,074	G	A	m	m	m	m	Francci3_3720	Peptide chain release factor 1	Leu206Phe
4,482,367	G	A	m	m	m	m	Francci3_3744	UvrD/REP helicase	Ala910Val
4,482,379	G	A	m	m	m	m	Francci3_3744	UvrD/REP helicase	Ala906Val
4,656,097	G	A	m	m	m	m	Francci3_3892	Hypothetical protein	Pro148Ser
4,859,393	A	G	m	m	m	m	Francci3_4061	TetR family transcriptional regulator	Stop196Trp
4,972,732	C	T	m	ND	ND	ND	Francci3_4168	DNA polymerase III subunit alpha	Gly67Arg
5,119,629	G	A	m	ND	m	ND	Francci3_4287	Hypothetical protein	Ala31Val
5,268,561	C	T	m	m	m	m	Francci3_4407	Dihydropteroate synthase	Gly132Asp
5,340,439	G	A	m	m	m	m	Francci3_4468	Hypothetical protein	Ala10Thr

[a] A mutation identical to that in mutant N3H4 was detected.

[b] The mutation found in mutant N3H4 was not detected.

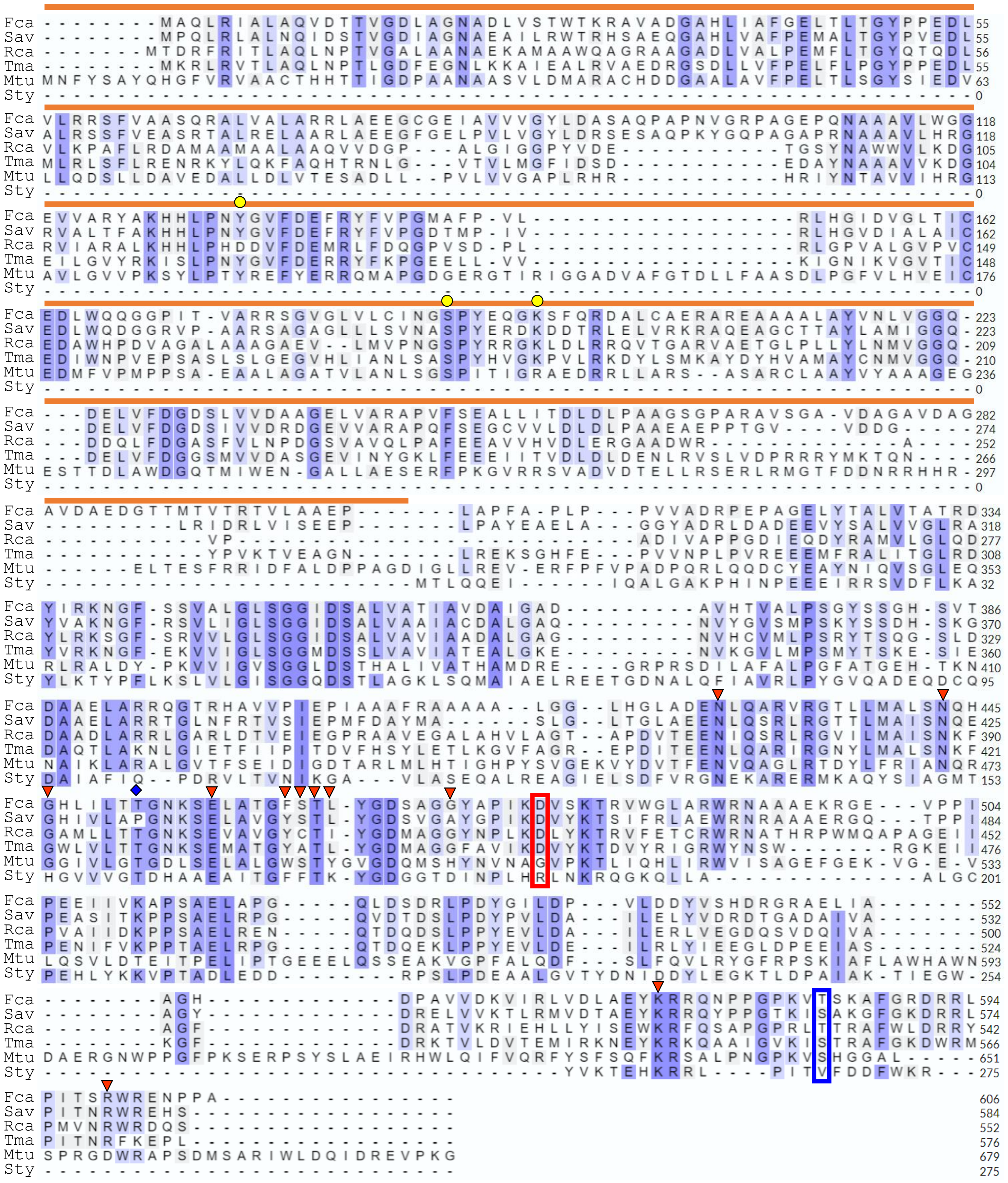

Fig. S1 Amino acid sequence alignment of NAD^+ synthetases. Sequences from *Frankia causarinae* (Fca), *Streptomyces avermitilis* (Sav), *Rhodobacter capsulatus* (Rca), *Thermotoga maritima* (Tma), *Mycobacterium tuberculosis* (Mtu) and *Salmonella typhimurium* (Sty) are shown. Binding sites for L-glutamine (yellow circles), nicotinic acid adenine dinucleotide (red triangles) and ATP (blue diamond) are shown. Orange line indicates the glutaminase domain. Amino acids corresponding to Asp478 and Thr584 in the *F. casuarinae* sequence are boxed.

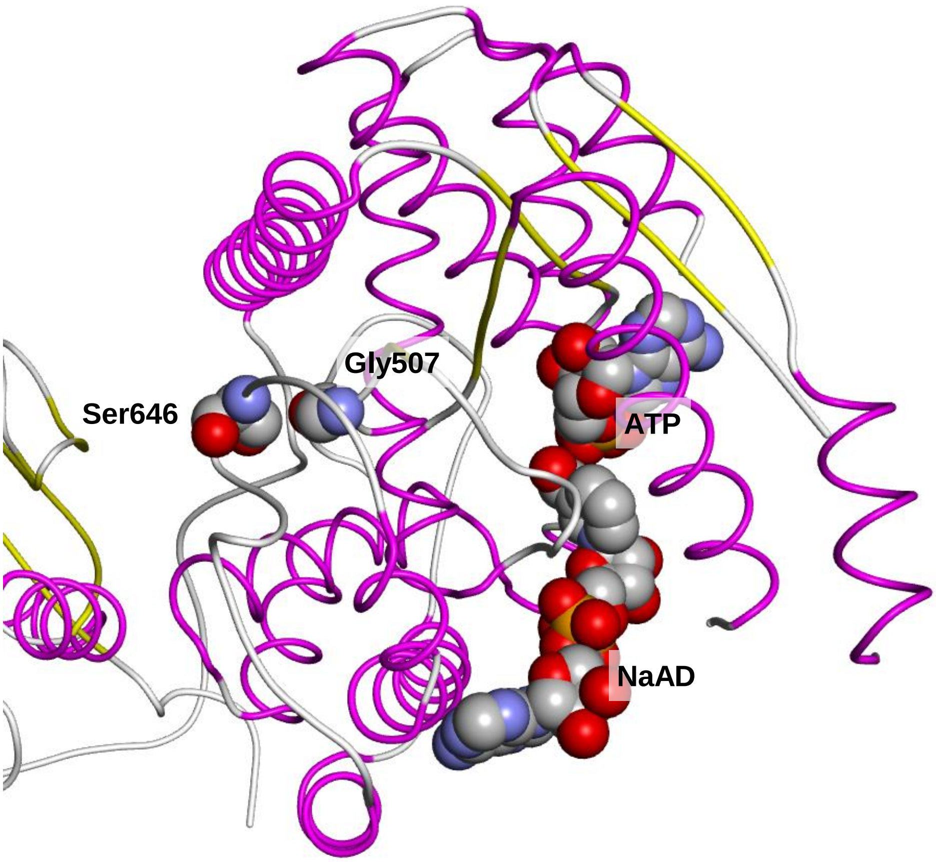

Fig. S2 X-ray crystal structure of NAD^+ synthetase from *Mycobacterium tuberculosis*. The Protein Data Bank accession number is 3SEZ. Gly507 and Ser646 correspond to Asp478 and Thr584 in the NAD^+ synthetase from *Frankia casuarinae* (Francci3_3146).
